# Burden and transmission dynamics of paediatric antimicrobial resistance in Benin City, Nigeria: a One Health study protocol

**DOI:** 10.1136/bmjph-2025-004340

**Published:** 2026-11-03

**Authors:** Kelly Osezele Elimian, Ephraim Ibadin, Jeremiah Igunma, Afamefuna Dunkwu-Okafor, Emmanuel Wemambu, Jonathan Inetianbor, Jasinia Musa, Ifeanyi Franklin Ike, Nina Viberg, Ayebo Evawere Sadoh, Tanya Monaghan, Tobias Alfven

**Affiliations:** 1 Department of Global Public Health, Karolinska Institutet, Stockholm, Sweden; 2 Department of Microbiology, University of Benin, Benin City, Nigeria; 3 GSOUTH Education, Abuja, Nigeria; 4 Medical Laboratory Services, University of Benin Teaching Hospital, Benin City, Nigeria; 5 Department of Medical Microbiology, University of Benin Teaching Hospital, Benin City, Nigeria; 6 Department of Applied Biosciences, School of Health, Science and Education, Coventry University Group, Coventry, UK; 7 Department of Veterinary Microbiology, University of Maiduguri, Maiduguri, Nigeria; 8 Trivision Technologies Limited, Abuja, Nigeria; 9 Institute of Child Health, University of Benin, Benin City, Edo state, Nigeria; 10 Department of Child Health, University of Benin Teaching Hospital, Benin City, Edo State, Nigeria; 11 Translational Medical Sciences, University of Nottingham, Nottingham, UK

**Keywords:** Epidemiology, Public Health, Qualitative Research, Cross-Sectional Studies

## Abstract

**Introduction:**

Antimicrobial resistance (AMR) poses a critical threat to global health security and disproportionately affects children in sub-Saharan Africa. In Nigeria, the burden of multidrug-resistant pathogens in paediatric populations is increasing; however, data from the South-South region, particularly Edo State, remain scarce. This study aims to determine the prevalence, patterns and transmission dynamics of AMR among paediatric patients in Edo State, Nigeria, using a One Health approach that integrates human, animal and environmental health interfaces.

**Methods and analysis:**

This mixed-methods study will enrol 455 hospitalised children (<18 years) at the University of Benin Teaching Hospital (UBTH). Clinical (urine, blood, rectal and nasopharyngeal swabs), environmental and animal samples will be collected to isolate and characterise locally recoverable bacterial pathogens prioritised with reference to the WHO 2024 priority pathogen list. Antimicrobial susceptibility testing will be performed using the Kirby-Bauer disc diffusion method in accordance with Clinical and Laboratory Standards Institute guidelines. Selected clinically or epidemiologically important resistant isolates will be stored at −80°C for possible future molecular characterisation, resources permitting. Key informant interviews with caregivers, healthcare providers and policymakers will explore the systemic enablers and barriers to paediatric AMR stewardship. Quantitative data will be analysed using descriptive statistics and logistic regression, while qualitative data will undergo thematic analysis guided by Braun and Clarke’s framework.

**Ethics and dissemination:**

Ethical approval has been obtained from the UBTH Health Research Ethics Committee (Ref: ADM/E 22/A/VOL.VII/14831247). Written informed consent will be obtained from all participants. Findings will inform antibiotic stewardship, strengthen surveillance and guide AMR policy at institutional, state and national levels. Results will be disseminated through peer-reviewed publications, policy briefs, institutional reports and engagement with national and international health agencies.

**WHAT IS ALREADY KNOWN ON THIS TOPIC**

- Antimicrobial resistance (AMR) is a leading cause of persistent infections and increased morbidity and mortality in children, especially in sub-Saharan Africa.
- One Health approaches are critical to understanding the interconnected transmission of AMR between humans, animals and environments.

**WHAT THIS STUDY ADDS**

- This study will generate the first comprehensive epidemiological data on paediatric AMR in Edo State, addressing a critical regional gap.
- By integrating clinical, environmental and animal sampling, it will provide novel insights into AMR transmission within hospital and household settings.
- Stored resistant isolates will support future molecular characterisation and local genomic epidemiology studies, resources permitting.

**HOW THIS STUDY MIGHT AFFECT RESEARCH, PRACTICE OR POLICY**

- Findings will inform locally tailored clinical guidelines for rational antibiotic use in paediatric care.
- Results will support evidence-based AMR surveillance strategies and policy interventions at state and national levels.
- Insights into systemic enablers and barriers will guide targeted AMR stewardship and behaviour change interventions among caregivers and healthcare professionals.

## Background

 The complex interaction network among micro-organisms and resistance determinants from diverse environments facilitates gene flow, thereby expanding antimicrobial resistance (AMR) across humans, animals and the environment, posing a major public health challenge.^1^ A coordinated multisectoral approach, such as One Health, is therefore essential. AMR causes persistent infections and increases morbidity and mortality; it remains a serious threat to global health, particularly for children who frequently require antibiotics for pneumonia, meningitis and urinary tract infections (UTIs).^2^ Predominant resistant pathogens in children include *Enterobacteriaceae*, β-haemolytic *Streptococcus* species, *Streptococcus pneumoniae*, *Staphylococcus aureus*, *Bordetella pertussis*, *Mycoplasma pneumoniae* and *Mycobacterium tuberculosis*.^3^ The lack of paediatric pharmacological data exacerbates inappropriate antibiotic use, especially in sub-Saharan Africa, where the burden of infectious diseases requiring both preventive and therapeutic antibiotics is high.^4–7^ Multidrug-resistant (MDR) pathogens, including extended-spectrum β-lactamase (ESBL)-producing *Enterobacteriaceae*, severely limit therapeutic options amid weak diagnostic infrastructure, poor laboratory quality control and limited epidemiological data.^8,9^ AMR surveillance remains fragmented and inconsistent, notably in Nigeria and other West African countries.^10^

Evidence from sub-Saharan African paediatric populations shows increasing AMR prevalence, with significant Gram-negative infections and resistant Gram-positive pathogens beyond the neonatal period.^3^ Nigeria mirrors these trends, facing rising AMR that worsens treatment outcomes and increases healthcare costs, morbidity and mortality.^11,12^ Sparse reports on paediatric bacteraemia in Nigeria are complicated by non-prescription antibiotic use and suboptimal diagnostics.^13^ Studies from northern states (Borno, Kano) report significant AMR in paediatric bacteraemia and urinary infections, particularly to ampicillin and cotrimoxazole.^14,15^ Similarly, nasal carriage of methicillin-resistant *S. aureus* (MRSA) among immunocompromised children indicates hospital-based risk factors such as antibiotic usage and hospitalisation.^16^ Southern states (Enugu, Abia, Oyo, Lagos) consistently report high resistance to first-line antibiotics, confirming widespread AMR in paediatric populations.^17–29^ Genotypic studies in Osun State identified key resistance genes and plasmids, highlighting the rapid evolution of resistant pathogens.^21^

Despite these data, a significant research gap in paediatric AMR persists in the south-south geopolitical zone, including Edo State. The lack of local epidemiological and microbiological data hinders targeted public health interventions. Evidence also links healthcare environments to MDR Gram-negative transmission among patients and workers.^30^ Although bacterial loads on hospital surfaces are low, most are contaminated with MDR organisms, posing risks to vulnerable patients.^31^ This study therefore aims to quantify and characterise the community-onset/admission AMR burden and transmission in children through a One Health approach that encompasses patients, hospital environments, and household animals in Edo State. Strategically located in Nigeria’s South-South region, Edo State’s dense population and high healthcare utilisation make it critical for AMR research. Findings will inform evidence-based policies, rational antibiotic use, and proactive surveillance and stewardship strategies. The study objectives are: (1) describe antibiotic prescription patterns; (2) isolate and phenotypically characterise resistant bacteria from patients, environments and animals; (3) determine prevalence and factors of resistant carriage; (4) store selected resistant isolates for future molecular characterisation, resources permitting and (5) explore systemic enablers and barriers to paediatric AMR stewardship.

## Materials and analysis

### Study setting

This study will be conducted in Benin City, Edo State, located in Nigeria’s South-South geopolitical zone. As of 2024, the state ranked 20th in population, with approximately 5 million inhabitants across 18 local government areas. Healthcare delivery involves federal, state and local governments, as well as a robust private health sector.

### Study design

The study will adopt a mixed-methods design, grounded in a One Health approach. One Health is an integrated, unifying approach that aims to sustainably balance and optimise the health of people, animals and environments.^32^ Objectives 1–4 will be addressed using a cross-sectional design, and objective 5 will employ qualitative key informant interviews to contextualise the microbiological findings and explore stewardship barriers and enablers. The cross-sectional study component will be reported according to the STROBE statement, while the qualitative component will be reported according to the Consolidated criteria for Reporting Qualitative research (COREQ) checklist—see the completed checklists in online supplemental materials 1 and 2.

### Study hospital and laboratory

The study will be conducted at the University of Benin Teaching Hospital (UBTH) in Benin City, Edo State. UBTH is a multispecialty, federal government-owned tertiary healthcare provider in Benin City (Southern Senatorial District). It has a 910-bed capacity and several clinical departments, including paediatrics and community health. The paediatric department admits about 120 patients per month. The hospital offers postgraduate training in paediatrics and employs 22 consultants, 8 registrars and 20 senior registrars. All laboratory analyses, including sample storage for future research (eg, genomic DNA samples), will be conducted at the UBTH laboratory, with potential genotypic analyses performed at the Nigeria CDC National Reference Laboratory in Abuja.

### Study participants

A pragmatic recruitment strategy will be adopted, enrolling patients based on a presumptive clinician diagnosis or, where unavailable, study staff assessment of antibiotic need. All hospitalised patients will be reviewed once weekly using a point prevalence survey approach. Inclusion criteria are age under 18 years (excluding neonates), provision of informed consent by a caregiver, assent from children aged 7–17 years and admission duration of less than 48 hours, to focus on community-onset/admission carriage and infections rather than infections arising during hospitalisation. Patients whose caregivers decline consent will be excluded. Routine clinical care will continue in accordance with hospital policy, with caregivers responsible for standard costs; the study will cover the expenses of any additional laboratory investigations required.

### Sample size and sampling

The sample size for the proposed study is estimated based on the formula proposed by Kirkwood and Sterne^33^


$$n=\frac{3.84\Pi (1-\Pi )}{w^{2}}$$

Where n=required minimum sample size; π=proportion of AMR in children and w=precision of estimate (ie, CI).

Assuming a 50% prevalence of resistant bacterial carriage among enrolled children (π=0.5) and 5% precision (w=0.05) at 95% CI, the required sample size is 384 children. This assumption is conservative in the absence of local paediatric carriage data from the study setting and is consistent with a study reporting high ESBL carriage among hospitalised children in sub-Saharan Africa.^34^ Allowing for a 15% non-response rate, the final sample size is 452 (~455) paediatric patients. Eligible participants will be selected by simple random sampling from hospitalised children who meet the inclusion criteria, using a computer-generated random sequence (eg, GraphPad) until the target sample size is reached. The sample size is modelled on carriage prevalence from rectal and nasal screening, not on pathogenic organisms isolated from clinically indicated urine or blood cultures. The animal component is exploratory and linked to enrolled children with resistant bacterial carriage or infection and reported animal contact; therefore, no separate animal sample size will be calculated.

### Cross-sectional study

A cross-sectional design will be used to investigate AMR among paediatric patients, hospital environments, and household animals within a One Health framework.

#### Priority pathogens

This study will use the 2024 WHO priority pathogen list as a guiding framework for prioritising clinically relevant bacterial pathogens detectable using available culture, biochemical identification and phenotypic antimicrobial susceptibility testing methods.^35^

#### Training

Before data collection, three microbiologists, three resident doctors and two veterinarians from the University of Benin will receive on-site training covering the study protocol, data capture via the Open Data Kit (ODK) app, sample collection and transport and quality assurance.

#### Data and sample collection

Eligible participants will be identified by trained data collectors (resident doctors), who will inform caregivers about the study and its ethical requirements through verbal and written explanations. Privacy will be maintained throughout, and clinical care will not be delayed. Caregivers will be given adequate time to provide informed written consent as per the study’s ethical protocol (online supplemental material 3). Data collection will commence in January 2026 to account for the ongoing industrial strike by medical practitioners across Nigeria and end in December 2026 (or attainment of the estimated sample size). Delays and rationale for data collection extension will be communicated to the ethics committee as needed.

Trained staff will administer piloted and pretested questionnaires with coded response options where possible (see online supplemental material 4) via the ODK App, review hospital records, monitor antibiotic prescriptions, perform brief physical examinations and collect clinical samples (urine, rectal, blood and nasal swabs). Rectal and nasal swabs will be obtained from all patients to assess gut and nasal carriage of resistant bacteria, at admission or within the first 48 hours of hospitalisation, while urine and blood samples will be collected only from those with suspected UTI or sepsis.^34,36^ Questionnaire variables will be grouped into the following domains: sociodemographic, healthcare exposure, antibiotic use, household environment, animal contact, clinical presentation, laboratory and stewardship.

##### Urine and rectal swab samples

Urine from toilet-trained children will be collected midstream; for others, via clean-catch or catheterisation. Samples will be placed in sterile containers and cultured the same day. Urine (10 μL) will be inoculated onto Cystine Lactose Electrolyte Deficient and Blood agar, incubated at 37°C for 24–48 hours, with ≥10⁵ CFU/mL indicating a UTI. Rectal swabs will be cultured on MacConkey agar with 5 mg/L cefotaxime at 37°C for 24 hours to screen for gut carriage of resistant Gram-negative bacteria. Lactose-fermenting Gram-negative rods will be identified using API 20E kits and preserved in Luria broth with 20% glycerol at −80°C for further analysis.

##### Blood samples

As many children may have received antibiotics before admission, blood cultures will be processed using the BacT/Alert automated system to enhance detection of bacterial and fungal growth. For infants, 1–2 mL of blood will be collected; for children >1 year, 2.5–5 mL of blood will be drawn into paired aerobic bottles containing glucose broth with sodium polyanethol sulfonate (SPS). Where the target volume cannot be safely obtained, the maximum feasible volume will be collected, the actual volume recorded, and the sample flagged as low-volume. No repeat venepuncture will be performed solely for research purposes. Bottles will be incubated for up to 5 days in the BacT/Alert instrument. Positive samples will be Gram-stained and subcultured on blood, chocolate, and MacConkey agars at 37°C for 24–48 hours. Colonies will be identified using biochemical tests (API Staph, API 20E, API Strep); *Haemophilus* spp. will be confirmed using X-discs and V-discs. Bacteraemia will be defined as the growth of at least one clinically significant organism. Coagulase-negative *Staphylococcus*, *Bacillus*, *Corynebacterium* or *Micrococcus* species will be considered contaminants unless consistent with clinical findings. Cerebrospinal fluid (CSF) will not be collected routinely or solely for research purposes. Where meningitis is suspected and CSF is collected as part of routine clinical care, CSF culture and antimicrobial susceptibility results will be abstracted as clinically indicated infection-related findings.

##### Nasal swabs

A saline-moistened cotton swab will be used to sample the anterior nares for *S. aureus*/MRSA carriage screening and transported in Stuart’s medium at room temperature. Within 2 hours, swabs will be inoculated onto mannitol salt and blood agar, then incubated aerobically at 37 °C for 24 hours. Golden-yellow, Gram-positive, catalase-positive and coagulase-positive colonies will be confirmed as *Staphylococcus aureus* using the API Staph test. Methicillin resistance will be determined using the Cefoxitin disc test according to Clinical and Laboratory Standards Institute (CLSI) guidelines.^37^ Confirmed MRSA isolates will be stored at −80°C for further analysis.

##### Animal samples

Veterinarians will collect blood and faecal samples from household animals (eg, dogs, cats, goats, sheep, cows, pigs, chickens, ducks) belonging to patients identified as carriers of resistant bacteria. Sampling will occur only in households that report contact with animals before hospitalisation, and the total number of animal samples will be fewer than the number of human samples. Where few animals are present, all eligible animals that have had recent contact with the child will be sampled. Where many animals are present, they will be prioritised based on frequency of contact with the child, species relevance and feasibility. From pets, specimens may include venous blood, faeces, urine, pus and swabs from the nasopharynx, wounds, ears and eyes. For livestock, approximately 4 g of faeces will be collected into vials with modified Cary-Blair transport medium. The main animal specimens will be faecal or rectal samples, with other specimens collected only where clinically or practically indicated. Composite pen-floor samples (0.5–1 g portions) will be pooled, mixed and similarly preserved. Nasopharyngeal swabs will be collected using sterile swabs, which will be broken into Cary-Blair media tubes after sampling. Environmental samples (soil, feed, drinking water) from animal surroundings will also be obtained.

##### Environmental samples

Hospital environmental sampling will target wet sites (eg, sinks, drains, toilets, wastewater) and dry, high-touch surfaces (eg, bed rails, tables, call bells) to describe environmental AMR reservoirs in the care setting, not to infer healthcare-associated acquisition or direction of transmission. Swabs will be taken using sterile commercial devices, and grab wastewater samples (50 mL) will be collected twice daily (10:00 and 14:00) from manholes in medical units at a depth of 10–15 cm. Samples will be transported on ice to the microbiology laboratory within 2 hours or refrigerated at 4°C until processing. Manholes will be disinfected with alcohol before sampling, and 3%–5% of each media batch will be incubated at 37°C for 24 hours to verify sterility and ensure data quality.

### Bacterial strain identification

Bacterial colonies from clinical and animal samples will be identified using standard microbiological techniques, including colony morphology, Gram staining and confirmatory biochemical tests. Members of the *Enterobacteriaceae* will be identified using the API 20E system (bioMérieux) per the manufacturer’s instructions. Catalase testing will differentiate *Staphylococcus* from *Streptococcus* spp., with catalase-positive isolates further identified using the API Staph kit. *Haemophilus* spp. will be confirmed using X and V factor discs, while Gram-positive diplococci will be verified with the optochin test on blood agar.^38^ Isolates requiring further confirmation will be stored for possible 16S rRNA sequencing, resources permitting.^38^

For wastewater samples, homogenised specimens will undergo 10-fold serial dilution in 0.85% saline. Aliquots (0.1 mL) will be plated on mannitol salt agar and MacConkey agar, each supplemented with 5 mg/L cefotaxime, and then incubated at 37 °C for 24 hours. *Staphylococcus aureus* will be confirmed via Gram staining, catalase and coagulase tests, and the API Staph kit; Gram-negative isolates will be identified using the API 20E system.^37^

### Extraction of genomic DNA

Genomic DNA may be extracted from selected stored bacterial isolates from clinical, animal and environmental samples, resources permitting. Either the standard phenol-chloroform method or commercial kits (eg, Qiagen DNeasy Blood & Tissue Kit) will be used for the extraction.

### Amplification of the 16S rRNA gene by PCR

For selected isolates requiring further confirmation, the 16S rRNA gene may be amplified by PCR using the universal primer pairs 27F/1492R or 1525R/63F.^39^ Positive and negative controls will be included. PCR conditions will follow Haro *et al*.^40^

### Sanger sequencing, bioinformatics, phylogenetic analysis

Selected 16S rRNA and resistance gene amplicons may undergo Sanger sequencing at a certified facility, resources permitting. Chromatograms will be analysed using BioEdit, with sequences aligned via ClustalW and identified by BLAST. Where feasible, phylogenetic trees will be generated in MEGA11 to compare selected isolates from human, animal and environmental sources.^41,42^

### Antimicrobial susceptibility testing

Antibiotic susceptibility will be determined on Mueller-Hinton agar plates using the Kirby-Bauer disk diffusion method^43^ as per the CLSI guidelines.^37^ Locally relevant antibiotics will be tested, and zones of inhibition interpreted as susceptible, intermediate or resistant.^37^ Resistance phenotypes will be determined primarily using phenotypic antimicrobial susceptibility testing. The following discs will be used for *Enterobacteriaceae*, *Acinetobacter* spp., *Pseudomonas* spp., *Staphylococci*, *Streptococci*, *Haemophilus* spp. and *Enterococcus* spp.: ampicillin (10 µg), meropenem (10 µg), imipenem (10 µg), ceftazidime (30 µg), ceftriaxone (30 µg), cefuroxime (30 µg), piperacillin-tazobactam (100–10 µg), levofloxacin (5 µg), amoxicillin-clavulanate (30 µg), gentamicin (10 µg), amikacin (30 µg), ciprofloxacin (5 µg), nitrofurantoin (300 µg), cefoxitin (30 µg), azithromycin (15 µg), erythromycin (15 µg), clindamycin (2 µg), doxycycline (30 µg), tigecycline (15 µg) and trimethoprim-sulfamethoxazole (1.25–23.75 µg). Quality-control strains, including *E. coli* ATCC 25922, will be tested in each batch.

### Determination of MDR, extensively drug-resistant and pandrug-resistant strains

Definitions by Magiorakos *et al*,^44^ will be applied: multidrug resistant (MDR) isolates are non-susceptible to ≥3 1 agent in ≥3 antimicrobial categories; extensively drug-resistant (XDR) isolates are non-susceptible to all but ≤2 categories; and pandrug-resistant (PDR) isolates are non-susceptible to all tested agents.

#### MRSA screening

All *S. aureus* isolates will be screened for methicillin resistance using a 30 µg cefoxitin disc and interpreted per CLSI, 2025 (35). Confirmed MRSA isolates will be stored at −80°C for further analysis.

#### ESBL and carbapenemase screening

All resistant Gram-negative isolates will be further screened for β-lactamase and carbapenemase production as follows.

ESBL screening: Isolates recovered from MacConkey agar containing 5 mg/L cefotaxime will be tested using the double-disc synergy method as described by Soltani *et al*.^45^ Bacterial suspensions adjusted to 0.5 McFarland will be inoculated on Mueller-Hinton agar, with amoxicillin-clavulanic acid (30 µg) placed centrally and ceftazidime (30 µg), cefotaxime (30 µg) and cefepime (30 µg) 20 mm apart. After incubation at 37°C for 18–24 hours, an expanded inhibition zone between the AMC and any cephalosporin disc will indicate ESBL production.

Carbapenemase screening: Isolates resistant to imipenem or meropenem will be tested using the modified carbapenem inactivation method per CLSI (2025). Briefly, 1 µL (*Enterobacteriaceae*) or 10 µL (*Pseudomonas aeruginosa*) of culture will be emulsified in 2 mL trypticase soy broth with a meropenem disc and incubated for 4 hours at 37°C. The disc will then be placed on Mueller-Hinton agar inoculated with *E. coli* ATCC 25922 (0.5 McFarland) and incubated for 18–24 hours. A zone diameter of 6–15 mm (or colonies within 16–18 mm) will indicate carbapenemase activity; ≥19 mm will be considered negative.

### Duration of data collection

Data collection will continue until the target sample size is reached or for up to 6 months, whichever comes first. Recruitment may end earlier if the sample size is achieved before the fourth month.

### Outcome variables and covariates

The primary outcome variable is the proportion of participants carrying phenotypically resistant bacteria from rectal and nasal carriage screening (eg, MRSA and MDR *E. coli*), including MDR, XDR and PDR strains, at admission or within 48 hours of hospitalisation. Resistance detected from urine and blood cultures will be reported separately among patients with suspected UTI or sepsis.

Secondary outcomes include antibiotic prescription patterns among participants; bacteriological profiles from all clinical samples; blood culture volume and low-volume blood culture status, where applicable; CSF culture and susceptibility findings where CSF is collected as part of routine clinical care; factors associated with antibiotic resistance (proportions and ORs for carriers vs non-carriers); and qualitative themes on enablers and barriers to paediatric AMR stewardship.

Study covariates are detailed in the questionnaire. Variables for the primary analysis will be selected based on their relevance to AMR carriage, including sociodemographic factors, recent healthcare exposure, antibiotic use, comorbidities, sanitation, water source, animal contact and household access to antibiotics. Symptom variables will be used mainly for clinical description and syndrome classification.

### Data management and analysis

Data will be downloaded from the ODK database, cleaned, validated and anonymised before analysis. Missingness will be summarised by variable. Missing covariate data will be handled using the missing indicator approach, with complete-case sensitivity analyses conducted where feasible. Isolates with intermediate or resistant results will be classified as non-susceptible.

Descriptive statistics will summarise participants and isolate characteristics by hospital, age, sex and specimen type. Carriage prevalence from rectal and nasal swabs will be reported separately from urine and blood culture findings. Questionnaire variables will be analysed according to the conceptual domains linking exposures to AMR carriage, infection-related outcomes and stewardship-related findings. Hospital environmental isolates will be analysed descriptively and interpreted as contextual One Health and infection prevention data. Proportions will not be calculated for categories with fewer than 10 isolates. Categorical variables will be compared using the χ^2^ or Fisher’s exact test, and continuous variables using the Student’s t-test or Mann-Whitney U test.

Factors associated with resistant bacterial carriage will be examined using univariable and multivariable logistic regression. Variables associated with the outcome at p<0.05 in univariable analysis, or deemed clinically or epidemiologically important, will be included in the multivariable model. A backward stepwise approach will be used to derive the final model, retaining variables at p<0.05 or where removal suggests important confounding. Multicollinearity will be assessed using variance inflation factors. Results will be reported as adjusted ORs (aORs) and 95% CIs. Analyses will be performed in STATA V.16.

### Potential limitations of the proposed methodology

This study has some anticipated limitations. First, due to resource constraints, molecular testing will not be performed routinely for all isolates, and the study will not attempt exhaustive molecular detection of all WHO 2024 priority pathogens or resistance genes. Although such an analysis would have enhanced understanding of AMR transmission within the One Health framework, clinically or epidemiologically important resistant isolates will be stored at −80°C for future molecular characterisation when resources permit. Second, different sample types may produce different prevalence estimates. Rectal and nasal swabs will estimate carriage among all enrolled children, while urine and blood cultures will reflect only suspected UTI or sepsis. Findings will therefore be reported by specimen type, and carriage prevalence will not be directly compared with infection-related culture prevalence. Third, the cross-sectional design will limit our ability to assess AMR’s clinical burden, as outcomes of resistant infections tend to vary with follow-up duration, with longer-duration studies reporting higher mortality rates than their counterparts with shorter follow-up (eg, postdischarge mortality vs in-hospital outcomes).^42^ Nonetheless, the planned qualitative component will complement the quantitative findings by providing contextual insights into the broader and non-quantifiable impacts of childhood AMR in the study setting. The animal sampling component is exploratory and conditional on household animal contact among children with resistant bacterial carriage or infection. It will therefore support One Health interpretation but will not estimate independent animal-level AMR prevalence. In addition, because participants will be enrolled within 48 hours of admission, the study will focus on community-onset/admission AMR carriage and infection. Hospital environmental sampling will provide contextual One Health data, but the study will not determine healthcare-associated acquisition or transmission during admission. Such questions would require a separate longitudinal study, including samples collected after 48 hours of hospitalisation or from recently discharged patients

### Qualitative data collection

Key informant interviews, guided by a social constructivist framework and semistructured interview guides, will explore the enablers and barriers to paediatric AMR stewardship and challenges surrounding childhood AMR. The qualitative outcome will be the main themes describing health-system, caregiver, clinical and policy-level barriers and enablers to paediatric AMR stewardship. The lead researcher (KOE), experienced in qualitative methods, will conduct interviews in the study hospital with at least 20 purposively selected caregivers (≥18 years), 20 healthcare providers (clinicians, pharmacists, nurses, laboratory scientists) and 10 policymakers (eg, hospital directors, Ministry of Health officials, Nigeria CDC AMR leads) until data saturation is observed. Potential participants will be approached using a mixture of face-to-face (during health-seeking or hospitalisation) and telephone (most likely for those recommended by colleagues or friends). Verbatim transcripts of audio-recordings will be analysed thematically using Braun and Clarke’s framework.^46^

#### Study monitoring and quality assurance

The study hospital will implement an internal data monitoring plan to ensure ethical compliance, data completeness and accurate sample handling. The study will adhere to the principles of the Declaration of Helsinki.^47^ The core research team will conduct periodic data audits, resolve queries and present progress reports to the study hospitals to maintain data quality and consistency.

#### Patient and public involvement

Patients and caregivers contributed to the study’s conceptualisation and design. Caregivers of hospitalised children helped refine data collection tools and ensure cultural appropriateness. Participants, community representatives and AMR stakeholders will be engaged during results dissemination.

#### Dissemination plan

Findings will be published in peer-reviewed journals and presented within participating institutions. A final report and policy briefs will be shared with national and international stakeholders, including the Nigeria Centre for Disease Control and Prevention, the Ministries of Health and Livestock Development and the WHO, among others. Results will also be presented at academic and community-focused conferences.

## Supplementary material

10.1136/bmjph-2025-004340 online supplemental file 1

10.1136/bmjph-2025-004340 online supplemental file 2

10.1136/bmjph-2025-004340 online supplemental file 3

10.1136/bmjph-2025-004340 online supplemental file 4

## Data Availability

Data are available on reasonable request.
