# Supplementary material for: Burden and transmission dynamics of paediatric antimicrobial resistance in Benin City, Nigeria: a One Health study protocol
Source: BMJ Public Health. 2026 Nov 3;4(4):e004340. doi: 10.1136/bmjph-2025-004340 (PMC13735319; doi:10.1136/bmjph-2025-004340)
Supplement: Supplementary file 2 [file bmjph-4-4-s002.pdf]

COREQ: Consolidated criteria for reporting qualitative research: a 32-item checklist for interviews and focus groups

Section/Topic	Item No	Checklist item	Reported on page No
**Domain 1: Research team and reflexivity**			
Personal Characteristics			
Interviewer/facilitator	1	Which author/s conducted the interview or focus group?Interviewer/facilitator	Yet to be known, as this is a protocol
Credentials	2	What were the researcher's credentials? E.g. PhD, MD	Available in the online submission system
Occupation	3	What was their occupation at the time of the study?	Available in the online submission system
Gender	4	Was the researcher male or female?	Both males and females
Experience and training	5	What experience or training did the researcher have? Relationship with participants	Yet to be known, as this is a protocol
Relationship with participants			
Relationship established	6	Was a relationship established prior to study commencement?	This is ongoing, but yet to be finalized
Participant knowledge of the interviewer	7	What did the participants know about the researcher? e.g. personal goals, reasons for doing the research	Yet to be known, as this is a protocol
Interviewer characteristics	8	What characteristics were reported about the interviewer/facilitator? e.g. Bias, assumptions, reasons and interests in the research topic	Yet to be known, as this is a protocol
**Domain 2: study design**			
Theoretical framework			
Methodological orientation and Theory	9	What methodological orientation was stated to underpin the study? e.g. grounded theory, discourse analysis, ethnography, phenomenology, content analysis	13 (lines 399-400)
Participant selection			
Sampling	10	How were participants selected? e.g. purposive, convenience, consecutive, snowball	13 (line 402)
Method of approach	11	How were participants approached? e.g. face-to-face, telephone, mail, email	13 (lines 405-406)
Sample size	12	How many participants were in the study?	Yet to be known, as this is a protocol
Non-participation	13	How many people refused to participate or dropped out? Reasons?	Yet to be known, as this

COREQ: Consolidated criteria for reporting qualitative research: a 32-item checklist for interviews and focus groups

			is a protocol
Setting of data collection	14	Where was the data collected? e.g. home, clinic, workplace	13 (line 402)
Presence of non-participants	15	Was anyone else present besides the participants and researchers?	Yet to be known, as this is a protocol
Description of sample	16	What are the important characteristics of the sample? e.g. demographic data, date	Yet to be known, as this is a protocol
Data collection			
Interview guide	17	Were questions, prompts, and guides provided by the authors? Was it pilot tested?	Yet to be finalised, as this is a protocol
Repeat interviews	18	Were repeat interviews carried out? If yes, how many?	Yet to be known, as this is a protocol
Audio/visual recording	19	Did the research use audio or visual recording to collect the data?	13 (line 407)
Field notes	20	Were field notes made during and/or after the interview or focus group?	Yet to be known, as this is a protocol
Duration	21	What was the duration of the interviews or focus group?	Yet to be known, as this is a protocol
Data saturation	22	Was data saturation discussed?	13 (line 405)
Transcripts returned	23	Were transcripts returned to participants for comment and/or correction?	Yet to be known, as this is a protocol
Domain 3: analysis and findings<br>Data analysis			
Number of data coders	24	How many data coders coded the data?	Yet to be known, as this is a protocol
Description of the coding tree	25	Did the authors provide a description of the coding tree?	Yet to be known, as this is a protocol
Derivation of themes	26	Were themes identified in advance or derived from the data?	Yet to be known, as this is a protocol
Software	27	What software, if applicable, was used to manage the data?	Yet to be known, as this is a protocol
Participant checking	28	Did participants provide feedback on the findings?	Yet to be known, as this is a protocol

COREQ: Consolidated criteria for reporting qualitative research: a 32-item checklist for interviews and focus groups

Reporting			
Quotations presented	29	Were participant quotations presented to illustrate the themes / findings? Was each quotation identified? e.g. participant number	Yet to be known, as this is a protocol
Data and findings consistent	30	Was there consistency between the data presented and the findings?	Yet to be known, as this is a protocol
Clarity of major themes	31	Were major themes clearly presented in the findings?	Yet to be known, as this is a protocol
Clarity of minor themes	32	Is there a description of diverse cases or a discussion of minor themes?	Yet to be known, as this is a protocol
