# Supplementary material for: Burden and transmission dynamics of paediatric antimicrobial resistance in Benin City, Nigeria: a One Health study protocol
Source: BMJ Public Health. 2026 Nov 3;4(4):e004340. doi: 10.1136/bmjph-2025-004340 (PMC13735319; doi:10.1136/bmjph-2025-004340)
Supplement: Supplementary file 3 [file bmjph-4-4-s003.pdf]

AMR STUDY QUESTIONNAIRE

Most variables will be captured using coded options in ODK, with ‘Other, specify’ retained where necessary. The questionnaire will be piloted before full data collection, and frequently repeated free-text responses will be converted into closed options to improve data quality and analysis.

SECTION 1 (Human; to be completed by a data collector)

Note: Variables in this section will be grouped into analytical domains. Core predictors will be used for AMR carriage analysis, while symptom, household and behavioural variables will support clinical description, One Health interpretation and stewardship objectives.

1. What is the hospital/health facility ID?
2. What is the patient’s name? __________
3. What is the patient’s location within the facility? Emergency / Non-emergency
4. Was the patient referred from another facility? Yes / No
5. If yes to Q3, facility name: __________
6. What is the patient’s sex? Male / Female
7. What is the patient's age? __________ month
8. What is the patient’s ethnicity? __________
9. What is the patient’s religion? __________
10. What is the patient’s residence setting? Rural / Semi-urban / Urban
11. What is the highest education attained by the caregiver? __________
12. What is the caregiver’s occupation? __________
13. What is the caregiver’s marital status? Single / Married / Divorced / Cohabiting
14. What is the caregiver’s average monthly income in Naira? __________
15. Does the child attend daycare (if under 5 years)? Yes / No
16. What was the primary reason for this hospital admission? __________
17. How many days did the illness last before hospitalisation? __________
18. Was the patient hospitalised in the past 6 months? Yes / No
19. Has the patient undergone surgery in the past 6 months? Yes / No
20. Did the patient have any indwelling medical devices (e.g., catheter, tube) in the past 6 months? Yes / No
21. Does the patient have any chronic skin conditions? Yes / No

22. Has the patient used any drugs for a sudden illness in the past 6 months? Yes / No
23. Has the patient used any drug for a recurring /chronic condition in the past 6 months? Yes / No
24. Does the patient have any co-morbidities (e.g., diabetes)? Yes / No
25. If yes, specify the comorbid condition(s)
26. Does the patient suck their thumb? Yes vs No
27. Has the patient experienced diarrhoea in the past 3 months? Yes / No
28. What type of toilet does the household use? Water system / Pit latrine / Open defecation
29. What is the main household water source? Piped / Borehole / Well / River/Stream
30. Has the patient had contact with any animals in the past 3 months? Yes / No
31. If yes, where was the contact made? House/ Farm/ Neighbour/ All of the above
32. How often does the patient have contact with animals? Daily / Weekly / Occasionally / Never
33. Has the patient or caregiver administered medication to animals? Yes / No
34. What is the first place for consultation when you or any member of your family fall ill? Chemist / Pharmacist /Family nurse /Family doctor /Primary health care centre /Private hospital/ Specialist public hospital
35. How often does the household buy antibiotics over the counter? Never, we buy from the hospital / 1-2 times/month / More than twice/month
36. Has anyone in the household had an illness that took a very long time to recover/heal (an indication of resistant infection)? Yes / No
37. Are antibiotics easily accessible without a prescription in your area? Yes / No
38. List all the signs/symptoms the patient had at admission (leave blank if the patient was asymptomatic or you can't identify the information; tick all that apply): Fever/cough/difficulty breathing / diarrhoea/vomiting / abdominal pain/dysuria/convulsion/rash/wound or skin infection / poor feeding/lethargy / other, specify: ________

SECTION 2: Human; to be completed by a data collector when an outcome for a patient is known)

1. Date of data collection

2. Patient unique ID:
3. What is the patient's diagnosis (combination of ≥2 diagnoses is allowed)?
4. What is the patient's outcome: Discharge/ Abandonment/ Death
5. How many days was the patient admitted (duration of hospitalisation)? ____
6. Have antibiotics been administered to the patient throughout the course of illness? Yes vs No
7. If yes to the previous question, specify the antibiotics:
    a. Antibiotic 1
        i. Antibiotic 1 class
        ii. Dosage of antibiotic 1 (mg per dose; calculate mg/kg where weight is available)
        iii. Route of administration of antibiotic 1
        iv. Frequency for each antibiotic 1 intake: ……….
        v. Duration of antibiotic 1 intake/treatment at the point of data collection (days)
        vi. Patient weight at prescription/admission: ____ kg

Calculated dose: ____ mg/kg/dose or ____ mg/kg/day, where availableRepeat the above antibiotic prescriptions x12

8. Were other interventions (drugs or procedures) given to the patient? Yes vs No
    a. If yes to the previous question, specify the intervention(s) (tick all that apply):
        i. oxygen therapy
        ii. intravenous fluids
        iii. blood transfusion
        iv. surgery
        v. wound dressing
        vi. catheterization
        vii. dialysis
        viii. other, specify: ________.
9. Duration of intervention: ____ days What is the type of treatment administered to the patient? Empiric/ Definitive/ Prophylactic

SECTION 3 (Animal, to be completed by the Veterinary team)

1. What type of animal sample is being collected from?
2. What is the animal's species and breed? __________

3. How old is the animal? __________
4. What is the animal's sex? Male / Female
5. What is the ownership status of the animal? Household / Farm / Stray / Wildlife
6. What is the primary purpose of the animal? Companion / Food production / Security / Other
7. Has the animal been exposed to farms or markets for more than 10 years? Yes / No
8. Has the animal had a recent illness (e.g., diarrhoea, respiratory signs)? Yes / No
9. Has the animal received antibiotics in the past 6 months? Yes / No
10. Has the animal been to a veterinarian clinic within the past year? Yes / No
11. Has the animal had contact with humans who have been diagnosed with any infection? Yes / No
12. Has the animal had contact with other sick animals? Yes / No
13. Animal sample type collected (tick all that apply):
    a. nasal swab
    b. rectal swab
    c. faeces
    d. blood
    e. urine
    f. wound swab
    g. ear swab
    h. eye swab
    i. environmental sample around animal
    j. other, specify: ________

SECTION 4 (Environment, to be completed by MSc student)

1. Where was the environmental sample collected? (Circle one) Hospital / Household / Farm / Market / Water source / Waste disposal / Other: __________
2. For hospital sampling, specify the location(s)
    a. Location 1:
    b. Location 2:
    c. Location 3:
    d. Location 4:
    e. Location 5:

3. What are the GPS coordinates of the sampling location (excluding the hospital)? __________
4. Is there evidence of open defecation or poor sanitation at the site? Yes / No
5. Is there animal waste at the site? Yes / No
6. Is there stagnant water at the site? Yes / No
7. Is there evidence of antibiotic or pharmaceutical disposal at the site? Yes / No
8. Is the site within 100 meters of livestock or animal markets? Yes / No
9. Type of environmental sample collected (tick all that apply):
    a. bed rail swab
    b. table swab
    c. sink swab
    d. drain swab
    e. toilet swab
    f. wastewater
    g. drinking water
    h. soil
    i. animal feed
    j. pen-floor sample
    k. other, specify: ________

SECTION 5 (Laboratory, to be completed by lab scientists for ALL samples collected)

What is the hospital/health facility ID? __________

What is the patient's unique ID? __________

What is the sample's unique ID?

What is the sample source? Human / Animal / Environmental

What type(s) of samples were collected?

1) For each sample, complete one row per isolate:
2) Sample ID
3) Sample type
4) Organism identified
5) Identification method
6) Antibiotic tested
7) Result: Susceptible, Intermediate, Resistant, or Not tested

8) ESBL phenotype: Yes-No-Not tested
9) Carbapenemase phenotype: Yes-No-Not tested
10) MRSA phenotype: Yes-No-Not applicable
11) MDR-XDR-PDR classification, if applicable

Sample antibiotic susceptibility test (antibiotic resistant profile)

No.	Antibiotic tested	Susceptible	Intermediate	Resistant	Not tested
1	Amikacin	☐	☐	☐	☐
2	Amoxicillin-Clavulanic Acid	☐	☐	☐	☐
3	Ampicillin	☐	☐	☐	☐
4	Azithromycin	☐	☐	☐	☐
5	Cefepime	☐	☐	☐	☐
6	Ceftazidime	☐	☐	☐	☐
7	Cefixime	☐	☐	☐	☐
8	Cefoxitin	☐	☐	☐	☐
9	Cefotaxime	☐	☐	☐	☐
10	Ceftriaxone	☐	☐	☐	☐
11	Cefuroxime	☐	☐	☐	☐
12	Ciprofloxacin	☐	☐	☐	☐
13	Clindamycin (Dalacin C)	☐	☐	☐	☐
14	Daptomycin	☐	☐	☐	☐
15	Erythromycin	☐	☐	☐	☐
16	Ertapenem	☐	☐	☐	☐
17	Gentamicin	☐	☐	☐	☐
18	Imipenem	☐	☐	☐	☐
19	Levofloxacin	☐	☐	☐	☐
20	Meropenem	☐	☐	☐	☐
21	Moxifloxacin	☐	☐	☐	☐
22	Nalidixic Acid	☐	☐	☐	☐
23	Nitrofurantoin	☐	☐	☐	☐
24	Ofloxacin	☐	☐	☐	☐
25	Piperacillin-Tazobactam	☐	☐	☐	☐
26	Rifampicin	☐	☐	☐	☐
27	Tetracycline	☐	☐	☐	☐
28	Tobramycin	☐	☐	☐	☐
29	Ticarcillin-Clavulanate	☐	☐	☐	☐
30	Tigecycline	☐	☐	☐	☐
31	Sulfamethoxazole-Trimethoprim	☐	☐	☐	☐
