# Supplementary material for: Burden and transmission dynamics of paediatric antimicrobial resistance in Benin City, Nigeria: a One Health study protocol
Source: BMJ Public Health. 2026 Nov 3;4(4):e004340. doi: 10.1136/bmjph-2025-004340 (PMC13735319; doi:10.1136/bmjph-2025-004340)
Supplement: Supplementary file 4 [file bmjph-4-4-s004.pdf]

# Study information and consent form

## Study information

**Title of the Study:** Burden and Transmission Dynamics of Paediatric Antimicrobial Resistance in Benin City, Edo State, Nigeria: A One Health Approach

**Introduction**
You are invited to participate in a study on antimicrobial resistance in children in Benin City, Edo State, caused by bacteria that have developed the ability to resist commonly used antibiotics. Before you decide to participate in this study, it is important that you understand why this study is being conducted and what it involves. Please take the time to read the following information carefully, and do not hesitate to contact our staff if any areas in this information sheet are unclear or if you would like more information. Contact information can be found in the last paragraph of this information sheet.

**Summary**
Antimicrobial resistance (AMR) refers to the ability of microbes like bacteria, viruses, fungi, and parasites to withstand the effects of antimicrobial drugs, making infections more difficult to treat. This resistance arises naturally as microbes evolve, but the misuse and overuse of antimicrobials accelerate it. When microbes become resistant, the once effective drugs become less or completely ineffective, potentially leading to longer infections, severe illness, and even death.

We understand you have a child or relative on admission to this hospital, and we want to assess the burden of AMR by taking clinical samples (e.g., urine, stool, blood, and nasal swab) for testing in the laboratory. The study will also help us understand how AMR is transmitted between patients and the environment, as we intend to screen the hospital environment for potential resistant bacteria. For children screened to carry resistant bacteria and have had contact with animals, we will also visit their homes to collect samples (e.g., urine and nasal swabs) from household animals (e.g., dogs and cats). The findings from the study, which covers humans, environment and animals (One Health approach), can help us learn how AMR is transmitted and develop better guidelines to support hospitalised children in the future.

The study is a collaboration between researchers from many institutions, including the University of Benin, University of Benin Teaching Hospital (UBTH), and Karolinska Institute.

<u>We would like to invite you to take part in the study if your child or relative:</u>
- Are under the age of 18, and
- Are willing to sign an informed consent

Participation in the study involves providing clinical samples for testing in the laboratory and completing a questionnaire. If you report owning or living with animals, you will also be asked if we can visit your home to take animal samples and discuss your interactions with them should your child or relative be found to carry resistant bacteria. **Participation in the study is entirely voluntary**. All the information we collect will be confidential and cannot be traced back to you.

**What does taking part in the study involve?**
Taking part in the study involves:

- Consent for clinical samples (e.g., urine, blood, stool and/or nasal swab) to be taken from your child or relative. A medical professional will collect the clinical samples and send them to the laboratory for testing for AMR. The test will help identify the best antibiotics to administer to your child or relative for improved treatment outcomes.
- Additionally, a 30-minute survey will ask questions about your socio-demographics and information about possible risk factors for AMR. The medical professionals may look for additional information about your child or relative from their medical records as needed.
- If your child or relative is found to carry resistant bacteria and you report having an animal (e.g., a dog or cat) at home, we would like to visit your home to take clinical samples from the animal for AMR testing. From the test, we could determine if there is a relationship between the resistant bacteria in your child/relative and those isolated from the animal. However, our study design cannot show causality (i.e., who infected whom and when).
- You may also be invited to participate in key informant interviews with the lead researcher to understand better the barriers and enablers of AMR stewardship or good practices.

**Is participation voluntary?**
Taking part in the study is entirely voluntary. You and your child/relative can only participate if you give written consent (or thumbprint as the case may be). You can change your decision at any time. If you decide that you and your child/relative no longer want to participate in the study before the end of the survey, **before providing clinical samples**, we will not use the data or material you have provided so far. You can choose not to answer a question at any time. If you decide to stop participating after data collection, we will remove your data from the database if it can still be traced back to you.
You do not need to give a reason for your decision not to take part in the study, to stop participation or not to answer specific questions. If you choose not to participate, you will not face any consequences.
You can only decide to give consent for the participation of your child/relative if you are over the age of 18.

**What are the risks of participating? What are the benefits?**
The individual risk and direct benefit of participating in the study are considered low. Participating in the study will require some time, but we will strive to keep it as brief as possible. A trained clinician or medical doctor will take the clinical samples from your child/relative. Minor problems may occur when taking clinical samples, such as discomfort, bruising, or bleeding. The nose swab may be unpleasant and irritating. The laboratory results will be available to you and will be communicated as soon as possible. This is the only instance when the data will be traced back to you.

All data will be stored and processed by the One Health Research Group in the Department of Microbiology at the University of Benin (Data Administrator), in line with standard procedures. However, the other project partners may access your anonymised data (i.e., without your personal identifiers, such as name, phone number, and address) for the purposes of this study only. We are unaware of any unlawful data processing by any project partners.

**What will happen if my child or relative tests positive for AMR?**
A positive clinical sample indicates that your child or relative is carrying bacteria resistant to certain antibiotics (don't worry—we will conduct a test to determine which antibiotic to use for treatment). If any clinical sample is positive after laboratory testing, we will report the test result to the medical team for further care according to routine management guidelines.

**Will others know that my child/relative and I are participating in this study?**
We will make every effort to keep your participation in the study confidential. During the survey, we will ask you to answer some personal questions about yourself, e.g. age, gender, profession, and religion. This information will not be connected to you on an individual level. It is collected to help the researchers describe the study participants in general and to facilitate comparisons with similar research. This means it is difficult to trace your answers back to you individually.

**Does the study cost anything? Will I receive compensation for taking part?**
Participating in the study is free.

**How will the data I provide be protected?**
Your personal data and that of your child/relative will be kept secret and **pseudonymised**. Your answers and contact details will not be directly connected during data analysis. All project team members are required to observe strict privacy rules, and your data is always kept secret. The Department of Microbiology at the University of Benin, responsible for data protection in this study, follows the Nigerian Data Protection Regulation procedures by the National Information Technology Development Agency. The processing of your data is based on your consent. The data only serves scientific purposes. Commercial use or transfer of your data to others is prohibited.
The data is shared among the research partners through a secure and password-protected server. Even with absolute care and many security measures, it can happen that someone steals or hacks your data. This risk is reduced through data security practices. These include controlling entry to buildings, controlling access to Tablets/computers, rules on sharing data, and other ways to avoid destruction or loss of data. Per the Nigerian Data Protection Regulation, data will be stored only for the period reasonably needed, e.g., 10 years before destruction. Our policy sets this time frame for good scientific practice.

The consent form below provides more information about your rights regarding the processing of personal data and your right to withdraw your consent at any time.

If you have questions about how data is protected, please contact the UBTH Health Research Ethics Committee at ubthresearchethics@gmail.com

**What do I need to do if I want to take part?**
Please read through the following page. If you agree to participate in the study, please tick the box at the bottom of the page to state your agreement.

**What do I do if I have questions?**
If you have any questions, please contact the research team at Kelly.elimian@uniben.edu or call +2348155465671

## Invitation and declaration of consent: Testing and survey

I ………………………………………………………………………………………………………
(first name and surname)
agree to participate in the study "*Burden and Transmission Dynamics of Paediatric Antimicrobial Resistance in Benin City, Edo State, Nigeria: A One Health Approach*".

I have been informed of the study's content, goal, risks and benefits. I have received clear information about the study and data protection in written form. I have understood the information given to me. I have had sufficient opportunities to ask any questions.

I understand that my data will be collected, stored, combined, analysed, or processed in electronic or other forms for 10 years and then destroyed.

I understand my data may be shared among the project partners, but only anonymously (e.g., without my name, address, phone number). I understand that the research team will take all reasonable efforts to keep my personal details private.

I understand that two of the project partners (Karolinska Institutet and the University of Nottingham) are outside Nigeria, where the Nigerian Data Protection Regulations do not apply. I consent to sharing my data with these institutions for the purposes of this study only.

I understand that I can withdraw my consent to participate in the study anytime. I do not need to give reasons. There will be no adverse impact. With my withdrawal, all processing and storage of my data will be stopped. The withdrawal is effective for the future. The use of my data will remain unaffected until I withdraw my consent.

I understand I have the following rights to my data as outlined in the Nigerian Data Protection Regulation:

- I have the right to request information about what data has been stored about me and my child/relative, and to have it corrected, completed, deleted, or restricted for processing. I also have the right to receive the data I and my child/relative have provided in a structured, common, machine-readable format. I can assert these rights as long as the data can be assigned to my person and/or my child/relative.
- I have the right to file a complaint with the UBTH Health Research Ethics Committee.

☐ I agree with the above statements and wish to participate in the study, which asks me about sociodemographic information, risk factors, and consent for my child/relative to have their clinical samples taken for laboratory tests.

☐ I agree with the above statements and consent for the research team to visit my house and sample animals as needed.

☐ I do not agree to participate in any part of the study.

## Invitation and declaration of consent: Key Informant Interview

We also invite you to participate in an interview about your experiences as a caregiver to understand the enablers and barriers to AMR stewardship.

We might contact you if you are interested in participating in an interview. Therefore, we ask for your contact details. The details will be collected in a separate database and cannot be attached to your individual answers in the survey in any way. This information will be stored in a secure location only accessible by the research team and will only be used to invite you to take part in the interview. They will be deleted or destroyed after the study. This can be up to two years after you have given your information.

If you provide your contact information, this does not mean you will automatically be invited to participate in the interview. Participants will be randomly selected.

☐ I would like to be invited to participate in an interview on AMR stewardship.

☐ I do not want to participate in an interview.

Date and place of signature:______________________________________________

Name of participant:____________________________________________________
*(First name and surname)*

Signature of participant:_________________________________________________

| |
|---|
| *To be filled in by the Interviewer:*<br>*I hereby declare that I have informed the participant on …………………. (date) about the nature, significance, and risks of the study, both orally and in writing. I have answered all questions, and the participant has been given a copy of the study information and consent form.*<br><br>*Name of Interviewer: ……………………………………………………………………….*<br>*(First name and surname)*<br>*Signature of Interviewer: ……………………………………………………………………* |

## Invitation and declaration of consent: Household visit

<u>We also like to visit your household.</u>

A team composed of veterinarians will visit your household. The veterinarians will test your domestic animals and pets (if you have them) and ask questions about how you interact with animals (even if you don't). You don't need to prepare anything for this visit.

We might contact you if you are interested in participating in a household visit. Therefore, we ask for your contact details. The details will be collected in a separate database and cannot be attached to your individual answers in any way. This information will be stored in a secure location that is only accessible by the research team. After the study, the data will be deleted or destroyed. This can be up to two years after you have given your information. The contact details are only used to contact you to facilitate the household visit.

If you provide your contact information, you will not automatically be invited to take part in the household visit. The researchers will identify potential participants based on the laboratory results for their child/relative and invite them to the study.

☐ I <u>would like to be invited</u> to participate in a household visit, which involves allowing my domestic animals to be tested and taking part in conversations.

☐ I do <u>not</u> want to take part in a household visit.

Date and place of signature:_______________________________________________

Name of participant:_________________________________________________________
*(First name and surname)*

Signature of participant:_____________________________________________

| |
|---|
| *To be filled in by the researcher:*<br>*I hereby declare that I have informed the participant on …………………. (date) about the nature, significance, and risks of the study, both orally and in writing. I have answered all questions, and the participant has been given a copy of the study information and consent form.*<br><br>*Name of Interviewer:* …………………………………………………………………………….<br>*(First name and surname)*<br>*Signature of Interviewer:* ………………………………………………………………………… |
